# COVID-19-Related Stressors and Mental Health Among Chinese College Students: A Moderated Mediation Model

**DOI:** 10.3389/fpubh.2021.586062

**Published:** 2021-06-18

**Authors:** Zaichao Han, Xiujuan Tang, Xiaoshan Li, Youtian Shen, Li Li, Jingjing Wang, Xiaowei Chen, Zhijun Hu

**Affiliations:** ^1^College of Life Sciences, Jiangxi Normal University, Nanchang, China; ^2^School of Psychology, Jiangxi Normal University, Nanchang, China; ^3^Center of Mental Health Education and Research, School of Psychology, Jiangxi Normal University, Nanchang, China

**Keywords:** COVID-19-related stressors, coping, mental health, perceived risk of being infected with COVID-19, online learning satisfaction

## Abstract

This study aims to examine the relation between COVID-19-related stressors and mental health among Chinese college students during the pandemic outbreaks, and the possible mediator or moderator between them. Five hundred and fifty Chinese college students were invited to complete an anonymous survey, and the data were analyzed with SPSS 16.0 software. The results shows that the number of stressors has a negative direct and indirect (through risk perception of being infected with COVID-19 disease) impacts on college students' mental health. Adaptive coping is a protective factor of students' mental health, and could be regarded as a buffer that attenuates the negative effect of the COVID-19-related stressors on risk perception of being infected with COVID-19 disease (or mental health). With regard to demographic variables, females, junior and senior students, or students whose family residence was worst hit by the pandemic tend to report poorer mental health during the pandemic outbreak. These findings enrich our understanding about the impact of the COVID-19 pandemic on college population and have implications for university counseling services during times of acute, large-scale infective disease outbreaks.

## Introduction

COVID-19 pandemic not only caused damage to individual physical heath, but also have a negative impact on individual mental health (1, 2). Recently, a study of 1120 respondents from 194 cities in China showed that 53% respondents reported a moderate or severe psychological impact, and approximately 30.3% (36.4%) respondents reported the symptoms of depression (anxiety) during early COVID-19 outbreak (3). Although psychological symptoms are common phenomenon for people lived in infectious areas, the significant individual differences in susceptibility to mental health problems was also found during the pandemic outbreak (4, 5). The student population (including college students) became susceptible to the pandemic and may easily develop mental health problem due to their immaturity of psychological development, and the fluctuation of the mood (6). In addition, taking online course was often seemed as one of challenge issues for college students during the pandemic, and student suicide related to online schooling was reported in many countries or areas (7, 8). However, recent researches related to the pandemic mainly focused on patients, front-line health care workers, or abnormal response to the pandemic among general population, we know little about the effect of pandemic on college student population and how these influences occur. Therefore, studying the impact of COVID-19 pandemic on college student population can enrich our understanding about the impact of pandemic on general population and holds the promise of information for counselors to prevent negative psychological effect of pandemics on the general college student population.

Stress-health theory believes that stressful events might cause individual physical or mental health problems (6). As a new infectious disease, highly contagious and harmful, rapid development, and no effective drugs for preventive or treatment are main characteristics of the COVID-19 pandemic (1). During the pandemic outbreak, students' mental health was influenced by some stressful issues related to the pandemic, including individual physical health under the threaten of the COVID-19 disease, individual daily routine disrupted, and so on. Those issues above are regarded as the COVID-related stressors in the present study. Previous research has shown that the large number of stressors related to infectious disease is a risk factor in predicting individual psychological symptoms during SARS (9). In addition, the risk perception of being infected with COVID-19 disease is the common response by general population during the pandemic (10, 11). The risk perception model of health suggests that stressful events related with infectious disease have indirect negative effects on individual mental health by increasing individuals' perceived risk of being infected with diseases (12). Thus, the first hypothesis was proposed as follows:

Hypothesis 1: Individuals who experience more COVID-19-related stressors might have a higher perceived risk of being infected with COVID-19, leading to worse mental health.

Coping refers to an individual's constantly changing cognitive and behavioral efforts when dealing with environmental or physiological challenges (13). It is often regarded as a moderator to explain individual differences in susceptibility to mental health problems during the pandemic. On one hand, individuals who use adaptive coping strategy frequently tend to engage in problem solving and take positive measures (e.g., wearing masks for outdoor activities) to protect them from harm in a dangerous world (2, 12), which may reduce the impact of stressors on the risk perception of being infected with COVID-19 disease. On the other hand, individuals who use adaptive coping strategy frequently tend to make great effort (e.g., taking medical treatment; receive psychological intervention) in dealing with the potential adverse consequence of being infected with disease (e.g., physical harm) (2), which might reduce the negative effect of the risk perception of being infected with disease on mental health. Previous research has shown that adaptive coping (e.g., problem-based coping) could be regarded as a buffer in the stress-psychological symptoms relationship (9). Thus, we proposed the second hypothesis as follows:

Hypothesis 2: Adaptive coping could serve as a buffer by attenuating the negative effect of stressor on perceived risk of being infected with COVID-19, and the negative effect of risk perception of being infected with COVID19 on individual mental health.

## Method

### Participants

During the pandemic outbreak, all college students in mainland China were asked to stay at home to take online course instead of offline course. A total of 550 students from the College of Life Sciences at Jiangxi Normal University took part in our health survey in April 15–19, 2020. With the help of university counselor, the survey was sent to students by email, and participants were asked to complete an anonymous, self-reported questionnaire and to return it by the deadline. In the process of data collecting, all researchers including university counselors received the online training, including the standard procedure of data collection, data confidentiality, and so on. Questionnaires with more than 15% of the items unanswered were excluded from the later analysis. Finally, the data of 522 students was used in the following analysis. The response rate was 95%. This study was approved by the ethics committees of the School of Psychology, Jiangxi Normal University, and written informed consent was obtained by all participants.

### Measurement

#### Mental Health

Mental Health Inventory (MH-5) (14) were used to measure the mental health of Chinese college students. The participants were asked to respond on a six-point Likert scale (1 = none of the time, 6 = all of the time). Higher scores indicate better health. The MH-5 has been proven to be adequate as a screener for depression symptoms or generalized anxiety disorder, and as a valid scale in predicting individual mental health among Chinese samples (15). In the current study, the Cronbach's alpha of the scale was 0.81.

#### COVID-19-Related Stressors

According to Main, the stressors related to infectious disease could be divided as following six groups: self-related events, family-related events, friend-related events, acquaintance-related events, information-related events, and other infectious disease related events (9). Main's view on stressor categorization of infectious disease was supported in our interview survey during COVID-19 pandemic. Therefore, we developed a checklist to assess participants' experience with COVID-19-related stressors based on the Main's categories of stressors related to infectious disease. The COVID-19-related stressors were grouped into six categories: (a) self-related events (three items, e.g., You have the experience of contacting with a confirmed COVID-19 case); (b) family-related events (three items, e.g., A member of your family was a confirmed COVID-19 case”); (c) friend-related events (three items, e.g., A close friend of yours was diagnosed with COVID-19); (d) acquaintance-related events [three items, e.g., Someone you know (not including your family members or close friends) had COVID-19-like symptoms (fever, coughing)]; (e) information-related events (two items, e.g., You heard others talking about the severity and contagiousness of COVID-19); and (f) other COVID-19-related events (two items, e.g., Your family members had to be in contact with others because of work). A score of 1 indicates that the participants has experienced the stressor related to COVID-19 during the pandemic, while a score of 0 indicates that they have not. With total score of all items computed, the high scores indicate that students experienced more COVID-19-related stressors.

#### Risk Perception of Being Infected With COVID-19

Based on risk perception with SARS (16), we developed two items to assess college students' perceived risk of being infected with COVID-19. The participants were asked to report their potential exposure to infected patients or their perceived chance of being infected with COVID-19 (very low, low, medium, high, or very high). A high mean score of these two items indicates that the respondent has a higher perceived risk of being infected with COVID-19.

#### Adaptive Coping

The Brief Coping Strategy Scale (17) was used to assess how frequently participants used coping strategies during the COVID-19 pandemic. The questionnaire includes problem-based coping (e.g., active coping) and emotion-based coping (e.g., denying). Participants responded to each item on a four-point scale (1 = *I have never used it*, 4 = *I always used it*). The difference between problem-based coping and emotion-based coping was used to measure individual coping during the pandemic. A higher score indicates that individuals tend to use adaptive coping (e.g., focus on problem solving) rather than maladaptive coping (e.g., escape). The Brief Coping Strategy Scale has been proven to have high reliability and validity for Chinese samples, and the Cronbach's α values of problem-based coping and emotion-based coping were 0.86 and 0.87 in the present study, respectively.

#### Social Demographic Variables

In the present study, social demographic characteristics include gender (male, female), grade, and family residence. For variable of family residence, Hubei province was regarded as a worst-hit area during the pandemic, while other areas were regarded as low risk zones.

### Data Analysis

Data analysis was conducted via SPSS statistical software version 16.0. First, the demographic characteristics of the college students was calculated for all variables of interest. Second, multivariate tests were applied to compare mental health among college students by groups. Third, multiple regressions were used to analysis the influencing factors of MH5 among college students during the pandemic. In regression model, mental health was regarded as an outcome variable, COVID-19-related stressors as a predictor, risk perception of being infected with COVID-19 disease as a mediator, coping as a moderator, and sex, grade, family residence as control variables. The moderator was mean-centered, as suggested by Aiken and West (18). There was no interaction term for the demographic variables (e.g., gender) in the final regression because there was no effect of the interaction of demographic variables and the predictor (or moderator or mediator) on mental health in the preliminary analysis. Multicollinearity was not considered a problem because the variance inflation factors for all terms in the models did not exceed the cutoff value of 7 (19).

## Results

### Descriptive Statistics

The demographic and characteristics of the study population are shown in Table 1. Among the 522 responding participants, 115 (22%) were male, 355 (68%) were middle grade (includes freshmen and sophomore), 167(32%) high grade students (include juniors and seniors), 47(17.8%) students whose family residence lived in Hubei province (The worst-hit area during the pandemic).

**Table 1:** Demographic characteristics of the college students by psychological symptoms.

		**MH-5**		**Stressor**		**Risk perception**		**Adaptive**	
**Characteristic**	***N*** **(percent)**	**M** **±** **SD**	***P***	**M** **±** **SD**	***P***	**M** **±** **SD**	***P***	**M** **±** **SD**	***P***
Observations	522(100)	4.76 ± 0.68		2.01 ± 1.44		1.43 ± 0.63		18.8 ± 6.27	
Gender			<0.05		0.28		<0.001		0.739
Female	407(77.9)	4.73 ± 0.64		2.04 ± 1.42		1.48 ± 0.62		18.80 ± 6.16	
Male	115(22.1)	4.87 ± 0.79		1.88 ± 1.53		1.23 ± 0.51		19.02 ± 6.68	
Grade			<0.05		<0.01		0.252		0.087
Middle	355(68)	4.81 ± 0.67		1.86 ± 1.45		1.39 ±0.58		19.20 ± 6.34	
High	167(32)	4.64 ± 0.68		2.33 ± 1.37		1.48 ± 0.64		18.10 ± 6.07	
Family residence			<0.01		<0.01		0.112		<.05
Low hit	426(82.2)	4.79 ± 0.65		1.96 ± 1.40		1.39 ± 0.57		19.06 ± 6.26	
High hit	47(17.8)	4.51 ± 0.71		2.55 ± 1.67		1.54 ± 0.74		17.02 ± 5.95	

### Status of Mental Health by Subgroups

Table 1 shows that female, high grade, or students whose family residence was worse hit by the pandemic reported poorer mental health than male, middle grade, or students whose family residence was slightly hit by the pandemic (*ps*. < 0.05). The results also shows that female students report higher risk perceptions of being infected with disease than male students (*p* < 0.05), and junior and senior students report more COVID-related stressors than freshman and sophomore (*p* < 0.05).

### Regression Analysis on the Impact of the Pandemic on Mental Health Among College Students

Regression analyses shows (see Table 2) that the number of COVID-19-related stressors negatively predicts mental health (β = −0.21*, p* < 0.001), while adaptive coping positively predict mental health (β = 0.59*, p* < 0.001) after controlling participants' gender, grade, and family residence. The number of stressors had an indirect negative effect on mental health through risk perception of being infected with COVID-19 disease [indirect effect: −0.03, CI (−0.05, −0.01)]. Hypothesis 1 was supported. The two-way interaction effect of coping and stressor on risk perception (β= –*0.17, p* < *0.05*) and mental health (β = *0.25, p* < *0.001*) is significant. The result of a simple slope analysis (16) shows that the number of stressors has a large impact on risk perception of being infected with COVID-19 disease at low level of adaptive coping (below −1 SD from mean) than that at high level (above 1 SD from mean) (*B*_low_ = 0.1*, p* < 0.001; *B*_high_ = 0.08*, p* < 0.05, *Z* = 6.8, *p* < 0.001). The number of stressors has an indirect negative effect (through risk perception of being infected with COVID-19 disease) on mental health [indirect effect: −0.08, CI (−0.14, −0.01)] at a low level of adaptive coping, instead of a high level. Hypothesis 2 was partially supported.

**Table 2:** Multiple regressions predicting psychological mental health from stressors, risk perception of being infected with disease and coping strategy (*N* = 522).

	**Mental health**		**Risk perception**		**Mental health**	
	**β**	**Δ *R*^**2**^**	**β**	**Δ *R*^**2**^**	**β**	**Δ *R*^**2**^**
Grade	−0.10^*^	0.03	0.05	0.04	0.01	0.44
Gender	−0.08		0.18^***^		−0.06	
Family residence	−0.12^**^		0.08		−0.03	
The number of stressors	−0.21^***^	0.39	0.28^***^	0.12	−0.19^**^	
Adaptive coping	0.59^***^		−0.20^***^		0.39^***^	
Adaptive coping × stressors	0.25^***^	0.02	−0.17^*^	0.01	0.25^***^	
Risk perception					−0.12^**^	0.01
Risk perception × coping					0.06	0.003

*Moderators were mean-centered before being entered into the equation. In all regression equations, participants' grade, gender, and family residence were regarded as control variables, β = standardized regression coefficient; stressors = COVID-19-related stressors; Risk perception = Risk perception of being infected with COVID-19 disease. For gender: 0 = male, 1 = female; grade: 0 = middle grade, 0 = high grade; family residence: 0 = the place was under low hit by the pandemic, 1 = the place was under high hit by the pandemic; Δ R^2^ = The added amount of total variance in the dependent variable captured by added predictors in the model*.

*** *p < 0.001*,

** *p < 0.01*,

* *p < 0.05*.

## Discussion

COVI-19 caused suffering for people who lived in infected areas. Recent researches related to the pandemic mainly focused on the epidemiology, clinical characteristics of infected patients, the genomic characterization of the virus, and psychological research of medical staff or patients (3–5), less attention was paid to the impact of the pandemic on college student population, and how these impacts occur. The present study contributes existed literature by investigating the relation between the COVID-19-related stressors and mental health among Chinese college students, and the possible mediator (risk perception of being infected with COVID-19 disease) and moderator (coping strategy) between them. It could enrich our understanding about the impact of the COVID-19 pandemic on college student population, and have implications for university counseling services during the pandemic.

Being similar to the result of previous study about the impact of SARS-related stressors on psychological symptoms (9), the COVID-19-related stressors had a positive relation with psychological symptoms. It indicates that students who experience a large number of COVID-19-related stressors tend to report more psychological symptoms. The possible reason might be that, when students experienced a large number of COVID-19-related stressors, students themselves or some important persons of them (e.g., friends) tend to expose to the COVID-19, and have high possibility to be infected with disease. For the awe of life, they tend to report more mental or physical problem (20). Besides, people who have experienced a larger number of COVID-19-related stressors might suffer more social influence of the pandemic, such as quarantine, social distancing, economic distress, changed daily life, and misunderstanding (e.g., illness stigma), which might be another reason for individual poor mental health during the pandemic (21–23). The mediated role of risk perception of being infected with COVID-19 disease provide an evidence for risk theory of health during the infectious disease outbreak (12). It highlights the important role of cognition in stress-health relationship during the COVID-19 pandemic, and provides an interpretation about the stress-health relation from risk perspective. That is, the large number of COVID-19-related stressors has indirect negative effects on college students' mental health through increasing individual (unreasonable) risk perception of being infected with disease. In addition, the result shows that adaptive coping can be a buffer in stress-mental health relationship or the relation of stress and risk perception of being infected with disease. It is consistent with previous study about the protecting role of problem-based coping in stress-psychological adjustment relationship during other infectious disease pandemics (e.g., SARS) (9). The possible reason might be that individuals who frequently use adaptive coping might take positive measure (e.g., wear mask for outdoor activities; receive psychological intervention) to change the unfavorable situation, which could protect them from being infected with disease or minimizing the negative impact of COVID-19-related stressor on their mental health.

For demographic variables, women tended to report a higher perceived risk of being infected with COVID-19 and poorer mental health. It could be explained by the white-male effect (24), which posits that males not only express less concerns about large scale of public health emergency (e.g., radiation from Fukushima) than women, but also interpret the emergency issue as a threat to financial stability rather than to physical well-being. For grade, junior and senior students reported worse mental health than students in other grades. The possible reason might be that, compared with freshmen and sophomores, junior and senior students are faced with the issues of graduation and job searching, and may experience more stressors and more psychological symptoms in the process of preparing for graduation or looking for a job (or internship). Students whose family residence was severely hit tended to report poorer mental health than those whose family residence was slightly hit by the pandemic. It was consistent with the previous research about the impact of family residence on psychological symptoms during other infectious disease pandemics (9). The possible reason might be that the physical health of students themselves (or some important persons around them) were more likely under threat of being infected with COVID-19 disease, if students' home was located in high risk areas.

The study has several limitations. First, the cross-sectional design does not allow the investigation of changes in individuals' mental health at different periods of the COVID-19 pandemic, which would provide a fuller picture of the psychological impact of the epidemic outbreak. Future research should consider a longitudinal design to examine the relationships among those variables above during the pandemic. Second, beside of the variables of coping and risk perception, the stress-adjustment relation might be influenced by other variables such as socioeconomic status, family influence and so on. More attention should be paid to those factors when discussing the stress-adjustment relation during the pandemic. Third, the study population might be biased due to convenient sampling, especially for its tendency toward female participants, which cannot estimate the representativeness.

## Conclusion

In this study, COVID-19-related stressor is a risk factor of mental health among college students. COVID-19-related stressors have a negative impact on college student's mental health through increasing the risk perception of being infected with disease, and adaptive coping can be regarded as a buffer in reducing the negative impact of COVID-19-related stressors on mental health (or risk perception of being infected with disease). Government should provide more approaches in reducing the negative impact of the COVID-19-related stressors on individual mental health (e.g., transmitting the knowledge of how to prevent and control COVID-19), and increase their confidence in fighting against the pandemic. And school manager should also pay more attention to the female, junior and senior students, or students whose family residence was severely hit by the pandemic.

## Data Availability Statement

The raw data supporting the conclusions of this article will be made available by the authors, without undue reservation.

## Ethics Statement

The studies involving human participants were reviewed and approved by the ethics committees of the School of Psychology, Jiangxi Normal University. The participants provided their written informed consent to participate in this study.

## Author Contributions

ZaH, XT, XL, YS, LL, JW, XC, and ZhH participated in the design of this study, carried out the concepts, design, data acquisition, analysis, manuscript editing, revising the manuscript critically for important intellectual contents, final approval of the version to be published, and agreement to be accountable for all aspects of the work. ZaH, XT, and XL have largest contribution for this manuscript. All authors contributed to the article and approved the submitted version.

## Conflict of Interest

The authors declare that the research was conducted in the absence of any commercial or financial relationships that could be construed as a potential conflict of interest.
